# Spent *Pleurotus ostreatus* Substrate Has Potential for Managing Fusarium Wilt of Banana

**DOI:** 10.3390/jof7110946

**Published:** 2021-11-09

**Authors:** Walter Ocimati, Evans Were, Anthony Fredrick Tazuba, Miguel Dita, Si-Jun Zheng, Guy Blomme

**Affiliations:** 1 Alliance of Bioversity and CIAT, Kampala P.O. Box 24384, Uganda; tazubatony@gmail.com; 2 Institute of Agricultural Sciences in the Tropics, University of Hohenheim, 70599 Stuttgart, Germany; e.were@uni-hohenheim.de; 3 Alliance of Bioversity and CIAT, Cali 763537, Colombia; m.dita@cgiar.org; 4 Alliance of Bioversity and CIAT, Kunming 650205, China; s.zheng@cgiar.org; 5 Alliance of Bioversity and CIAT, Addis Ababa P.O. Box 5689, Ethiopia; g.blomme@cgiar.org

**Keywords:** basidiomycetes, biocontrol, corm damage, *Fusarium oxysporum* f. sp. *cubense*, metabolites, spent mushroom substrates

## Abstract

A range of basidiomycetes including the edible mushroom *Pleurotus ostreatus* (*Po*) can suppress plant pathogens such as *Fusarium* spp. With the current increase in production and consumption of *Po* in Uganda, the spent *Po* substrate (SPoS) could be an alternative to manage Fusarium wilt of banana (FWB), caused by the soil borne pathogen *Fusarium oxysporum* f. sp. *cubense,* race 1 (*Foc*). This study determined the potential of SPoS to inhibit *Foc* in vitro and in potted plants. In vitro studies confirmed suppression of *Foc* in pure co-culture (*Po* vs. *Foc*) assays and media amended with different concentrations (0% to 50% *w*/*v*) of un-sterilized SPoS filtrates. *Foc* growth in the sterile SPoS filtrate was comparable to the water control, suggesting possible roles of biotic or thermolabile components of the SPoS. To further verify the suppressive effects of SPoS, pot experiments were carried out with a resistant (‘Mbwazirume’, AAA) and susceptible (‘Sukali Ndizi’, AAB) banana cultivar using both artificially and naturally infested soils. Independent of the inoculation method, SPoS significantly reduced the severity of FWB in pot experiments. Susceptible cultivar ‘Sukali Ndizi’ growing in substrates amended with SPoS showed lower (1.25) corm damage (Scale 0–5) than the un-amended control (3.75). No corm damage was observed in uninoculated controls. The resistant cultivar ‘Mbwazirume’, showed slight (0.25) corm damage only in the Foc-inoculated plants without SPoS. These findings suggest that SPoS could be used as part of the management practices to reduce the impact of FWB.

## 1. Introduction

The white rot fungus *Pleurotus ostreatus* is the second most important cultivated edible mushroom after *Agaricus bisporus* (white button mushroom) [1]. This fungus naturally lives as a saprophyte on dead or decaying wood and is cultivated as an edible mushroom using lignocellulosic wastes and agricultural by-products such as coffee husks, corn cobs and cotton seeds [2,3]. For every kilogram of mushroom, around 5 kg spent mushroom substrate (SMS) is produced [4]. After harvesting the fruiting bodies, the SMS is mostly discarded.

Edible mushrooms including *P. ostreatus* have been reported to play multiple roles in regenerative farming. The spent mushroom substrate (SMS) or spent mushroom compost (SMC) have a high organic matter content (22–40%), high nutrient levels, high cation exchange capacity and slow mineralization, making these substrates efficient for plant growth [5,6]. Indeed, mushroom hyphae have been reported to wrap around plant roots and increase water and mineral availability for plants, thus improving plant yields. In maize (*Zea mays)*, a 20% yield increase has been reported when grown together with *Stropharia rugoso-annulata*, and in Brussels sprout (*Brassica oleracea* var. *gemmifera*), a 25% yield increase has been reported when grown with *Hypsizygus ulmarius* [5,6].

The potential use of mushrooms as biocontrol agents of plant pathogens and crop pests has attracted considerable attention during the past few decades [7,8]. Mushrooms in the form of mushroom isolated compounds, the SMS, SMS extracts and SMC have been shown to affect plant pathogens. In the presence of abiotic or biotic stresses, such as interspecific interactions with other fast-growing fungi, *P. ostreatus* produces several secondary metabolites which have a wide range of physiological functions, including defense against invading microorganisms and survival under stressful conditions [9,10]. Mushroom extracts and isolated compounds, such as peptides and proteins, sesquiterpenes and other terpenes, steroids, organic acids and quinoline, have been shown to have antimicrobial activity [11]. During interspecific interactions with other fungi, the laccase activity of *P. ostreatus* has been associated with suppression of the growth of aerial mycelium, inducing severe cytological disturbances in several pathogenic fungi [12].

The SMS/SMC is composed of fungal mycelia, extracellular hydrolytic and oxidative enzymes secreted from mushrooms for the degradation of substrates, and unused, but partially modified, lignocellulosic substrates [13]. Spent mushroom compost tea was reported to have positive effects in the control of *Phytophthora capsici* and *Phytophthora parasitica* on pepper (*Capsicum annuum* L.) both in in vitro and in vivo assays [14]. The suppressive/antifungal effects of aqueous extracts of spent *P. ostreatus* substrate (SPoS) have also been reported against *Fusarium oxysporum* f. sp. *lycopersici* (*Fol*) in in vitro and in vivo studies [15]. The SPoS was also associated with diverse beneficial microorganisms including fluorescent *Pseudomonas* spp., *Trichoderma viridae*, *Baccilus* spp., *Penicillum* spp., and *Aspergillus terrus*, which showed strong antagonism to *Fol* [15].

The reports mentioned above suggest a suppressive effect of certain metabolites and microorganisms in the SPoS towards some plant pathogens. Suppression of *Fol* by SPoS also suggests that SPoS could potentially be used to manage *Fusarium oxysporum* f. sp. *cubense* (*Foc*), the causal agent of Fusarium wilt of banana (FWB) [15]. FWB has severely impacted banana production worldwide and is currently considered as one of the major threats of this crop [16,17,18]. In Uganda, *Foc* race 1 has hampered the production of dessert banana cultivars (e.g., ‘Gros Michel’, *Musa* AAA and ‘Sukali Ndizi’ (syn. ‘Ndizi’), *Musa* AAB) and the ABB beer types (e.g., ‘Pisang Awak’ and ‘Bluggoe’).

In infested soils, *Foc* can survive for decades, through its resistant spores (chlamydospores) or as an endophyte on alternative hosts [19]. Organic amendments enriched with beneficial microorganisms have already proven to boost soil suppressiveness and reduce FWB intensity [20,21]. Thus, the SPoS could also be explored as an alternative to manage FWB, yet this has not been investigated to date. This study explored the potential of *P. ostreatus* and SPoS to suppress *Foc* race 1 in vitro and in vivo on potted plants.

## 2. Materials and Methods

This study was conducted between 2016 and 2020 at the National Agricultural Research Laboratories (NARL) of the National Agricultural Research Organization, Kawanda in Uganda. The study comprised in vitro laboratory experiments and pot experiments in a greenhouse.

### 2.1. Fungal Strains, Culture Conditions, and Plant Material

Healthy looking fruiting bodies of *P. ostreatus* (Supplementary Figure S1) were obtained from MULTIMUSH Limited, a national mushroom research for development company in Uganda. Pure fungal colonies of *P. ostreatus* were obtained by culturing approximately 5 mm square tissue stubs excised from the fruiting bodies on potato dextrose agar (PDA; Difco Laboratories, Detroit, MI, USA) plates and maintained through regular sub-culturing. The most vigorous isolate of *P. ostreatus* was used for the subsequent studies.

The SPoS used in this study were obtained from a mushroom incubation facility hosted at NARL, Kawanda. The mushroom gardens were made of cotton seed waste. *Fusarium oxysporum* f. sp. *cubense* (*Foc*) was isolated from the rhizome of banana plants of the cv. ‘Ndizi’ (i.e., ‘Sukari ndizi’, *Silk,* AAB) showing typical Fusarium wilt symptoms at NARL, Kawanda (0.40522° N, 032.53283° E, 1182 m.a.s.l.), in central Uganda. The pathogen was cultured on PDA plates and incubated at 28 °C in the dark for 7 days and subsequently confirmed as *Foc* using species-specific PCR primers [22]. A single spore colony of the initial culture was inoculated into five test plants of ‘Ndizi’ and observed to cause characteristic above—and below—ground Fusarium wilt symptoms and subsequently used in the study.

Tissue culture derived banana plantlets having at least four functional leaves were obtained from a certified banana company—Agro-Genetic Technologies (AGT). Two banana varieties were used: ‘Ndizi’ which is highly susceptible to *Foc* [23] and ‘Mbwazirume’ (*Musa* AAA), an East African Highland (EAH) banana previously reported as resistant to *Foc* [24].

### 2.2. In Vitro Interactions of Pleurotus ostreatus and Fusarium oxysporum *f. sp.* cubense

The in vitro screening assays were performed in 90 mm Petri plates with PDA. Mycelial plugs of about 3 mm square were cut out with a sterile blade from actively growing mycelial edge of *P. ostreatus* and *Foc* and placed 70 mm apart on either ends of the same Petri dish. Controls containing only plugs of each fungus were simultaneously set up in separate Petri dishes (Supplementary Figure S2).

Petri dishes were then incubated at 28 °C and monitored for 30 days. Growth characteristics such as overgrowth, contact inhibition, and distance inhibition of the fungal organisms were visually observed, and growth was measured after every three days. The experiment was repeated four times, with four replicates per treatment in each experiment.

Stubs of 2 mm were cut out 12 days post inoculation (d.p.i.) from (i) the *Foc* only control plate, (ii) the *Foc*-*P. ostreatus* co-culture interaction zone, and (iii) the *Foc* end close to the *Foc*-*P. ostreatus* co-culture interaction zone, using a 2 mm Harris Micro-punch (Sigma-Aldrich, Merck KGaA, Darmstadt, Germany). The stubs were then broken down in 3 mL of sterile distilled water using a sterile metallic rod and vortexed (Scientific Industries, Inc, Bohemia, NY, USA) to form a fine suspension for enumeration of *Foc* inoculum. Each of the 2 mm stubs above acted as a treatment, and a total of 6 stubs were cut per zone. 1 mL of the original 3 mL suspension was serially diluted with distilled water to 10^−4^. The suspension was vortexed between each dilution step to ensure a homogenous suspension. A volume of 20 µL of the suspension of each dilution was put into each side of the hemocytometer groove, and the spores in zones A–E of both sides of the hemocytometer were counted. The above procedure was repeated in triplicates, and the total spore count per replicate was computed as below:

Spores/mL = (*n*) × the dilution used/0.02; where *n* = the average spore count per square.

### 2.3. Effect of Extracts from Spent P. ostreatus Substrate (SpoS) on F. oxysporum *f. sp.* cubense (Foc) Mycelia Growth

Assessment of the inhibitory effect of SPoS was conducted as described by Szczech [25] and Bernal-Vicente et al. [26]. Different air-dry weights (1 g, 5, 10, 15, 20, 25, and 50 g) of SPoS were mixed with 100 mL of double distilled water resulting in 0% *w*/*v*, 1% *w*/*v* to 50% *w*/*v* of SPoS concentrations. The mixture was vortexed for 10 min and left to stand at 28 °C for 48 h, after which it was vortexed again and filtered through sterile cheese cloth. Half of each filtrate concentration was sterilized in an autoclave at 121 °C and 15 psi of pressure for 15 min to destroy or denature any biotic components of the filtrate. PDA media prepared with a low water content was filled with the unsterilized and sterilized filtrates above in three replicates. Plates filled with sterile water only served as controls. Thus, there were a total of 15 treatments. Discs (5 mm) of 10-day old *Foc* cultures on PDA were then placed in the center of each plate and incubated at 28 °C. The plates were observed daily over a period of 30 days. Colony diameters of the *Foc* mycelia were measured daily from the second day over a period of 12 days. The experiment was repeated twice, with three replicates per treatment in each experiment.

### 2.4. Greenhouse Evaluation of Spent P. ostreatus Substrate (SpoS) against Fusarium oxysporum *f. sp.* cubense

The greenhouse experiment determined the effect of a one-time application of SPoS on FWB severity in potted tissue culture-derived plants. Pot experiments were conducted using pre-sterilized *Foc* inoculated soils and unsterilized naturally infested soils. Pots of 2.5 L volume, able to take up to 2.3 kg of fresh soil at full capacity were used for the experiments.

*Presterilized soil inoculations*: Loam soil collected from an area with no history of *Foc* was sterilized for this study. The pot treatments comprised (i) a control treatment in which soil was not inoculated with *Foc or* SPoS, (ii) soils inoculated with 80 g of SPoS (i.e., 30% *v*/*v* of the pots) only, (iii) soils inoculated with 50 g of *Foc* colonized millet grain only, and (iv) soils inoculated with a combination of 80 g SPoS and 50 g millet grain colonized by *Foc*. The mean number of *Foc* colony-forming units (cfu) in 1 g of *Foc* colonized millet grain was estimated to be approximately 7.6 million/g using the standard laboratory procedures described above. Effort was made to thoroughly mix soils with either *Foc* or SPoS to ensure a more homogenous mixture. For trials in which *Foc* was combined with SPoS, the *Foc* inoculum was allowed to establish for two weeks before the introduction of SPoS. The SPoS was also allowed to establish for two weeks before introduction of banana plantlets. Thus, the time from *Foc* inoculation to introduction of banana plantlets was 4 weeks and 2 weeks from addition of SPoS to introduction of banana plantlets. Plantlets of two banana genotypes, a susceptible cultivar ‘Ndizi’ and a resistant cultivar ‘Mbwazirume’, were used in this study. Four plants per genotype, each acting as a replicate, were used per treatment, and the experiment was repeated twice.

*Naturally infested soils*: Naturally infested soils (mean *Foc* cfu = 0.53 × 10^6^ g^−1^) obtained from a hotspot within the NARL research station were assessed to mimic field conditions where multiple factors potentially affect interactions between microbial organisms. The experiment comprised only the susceptible ‘Ndizi’ cultivar planted in pots filled: (i) with *Foc* infested field soils only, (ii) *Foc* infested field soils thoroughly mixed with 80 g of SPoS, (iii) pre-sterilized soil thoroughly mixed with SPoS, and (iv) pre-sterilized soil only. For treatments with *P. ostreatus*, banana plantlets were introduced two weeks after the addition of SPoS. The rest of the steps were as for the experiment using sterilized soils above. Four plants were used per treatment, each acting as a replicate.

*Data collection*: The potted plants were regularly watered and monitored for a period of 2.5 months. Data collected from the pot trials included plant girth at soil level and height, the time to first foliar symptom expression, foliar symptom incidence (i.e., number of infected plants), and severity and corm damage score. A modified corm damage score (scale 0 to 5; 0 = no disease and 5 = severe corm damage) and leaf symptom severity score (scale 0 to 5; 0 = no yellowing of leaves and 5 = dead plant) developed by Viljoen et al. [27] was used.

### 2.5. Data Analysis

In the in vitro culture studies, the growth of the two fungi were compared on the co-culture plates and individual plates to ascertain suppressive effects from either fungus using analysis of variance (ANOVA) computed using the R statistical package [28]. The numbers of *Foc* spores in the different zones of the co-culture plates and *Foc* control plates were also compared.

The mean values of the diameters of *Foc* mycelia growth in SPoS extracts (sterilized and unsterilized) and controls of the in vitro studies were also subjected to ANOVA. Mean values of the data collected from the greenhouse pot trials for the different variables were analyzed using ANOVA. Visualizations of data were implemented using the R package ‘ggplot2′ [29].

## 3. Results

### 3.1. In Vitro Interaction between Pleurotus ostreatus and Fusarium oxysporum *f. sp.* cubense

**Figure 1:**
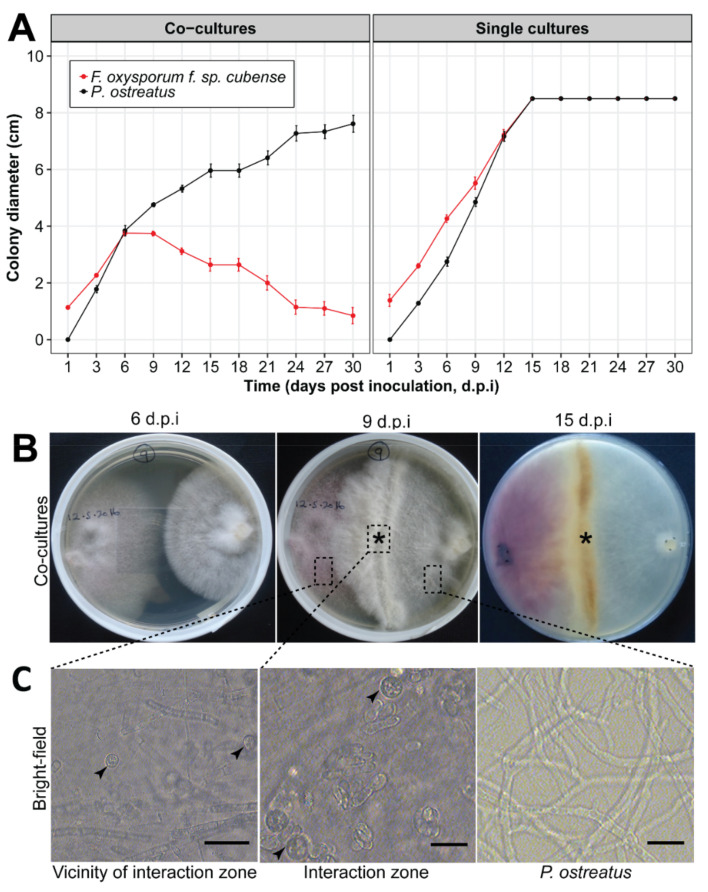
(**A**) Mycelia growth of co-culture and sole cultures for *Fusarium oxysporum* f. sp. *cubense* (*Foc*) and *Pleurotus ostreatus* (*Po*). (**B**) In vitro *Po-Foc* co-culture plates at 6-, 9-, and 15-days post inoculation (d.p.i.). (**C**) Microscopic images (40×) from three zones of the *Foc-Po* co-culture plate showing *Foc* zone next to the *Po*-*Foc* interaction zone, the *Po*-*Foc* interaction zone, and the *Po* zone. The asterisk (*) show thickened *Po* mycelia at 9 d.p.i. and subsequent denaturing of *Foc* mycelia at 15 d.p.i. Black arrows show Foc chlamydospores. Bars represent 1 µm magnification.

Both, *Foc* and *P. ostreatus*, showed similar growth patterns on solid PDA media in single cultures (Figure 1A, right panel). However, dual cultures of *P. ostreatus-Foc* showed strong interactions starting at the 6th d.p.i. when the two fungi came into contact. At that time-point, *P. ostreatus* hyphae were observed to thicken at the point of contact with *Foc* mycelia, followed by development of an inhibition zone (Figure 1B, middle panel). After 9 d.p.i., the thickened *P. ostreatus* mycelia, though at a slower rate compared to the single *P. ostreatus* control cultures, were observed to grow over and into the hyphae growing zone of *Foc*. At that time-point *Foc* hyphae turned brown and seemed to gradually degenerate in the zone of interaction (Figure 1A left panel and Figure 1B middle and right panels).

At 12 d.p.i. a significantly (*p* < 0.05) lower number of *Foc* spore counts (11.7 × 10^3^) was obtained in the *P. ostreatus-Foc* interaction zone when compared to the zone in the vicinity of the point of interaction between *P. ostreatus* and *Foc* (68.7 × 10^3^ spores) and the *Foc* single culture control (117.5 × 10^3^ spores). Micro and macro-conidia dominated the *Foc* culture plates. However, samples collected from *P. ostreatus-Foc* interactions zones and their vicinity (co-cultured plates) were dominated by *Foc* chlamydospores, with a lower density observed in the *P. ostreatus-Foc* interaction zone (Figure 1C).

### 3.2. Effect of SPoS Extracts on Fusarium oxysporum *f. sp.* cubense Growth

Mycelia of *Foc* growing on PDA amended with autoclaved SPoS filtrate covered entire plates. In contrast, *Foc* growth was restricted and limited in plates with the unsterilized SPoS filtrate. The mycelia of *Foc* in these plates turned brown and appeared to have degenerated as for the zone of interaction of the *P. ostreatus*-*Foc* co-culture plate above (c.f. Figure 1B).

**Figure 2:**
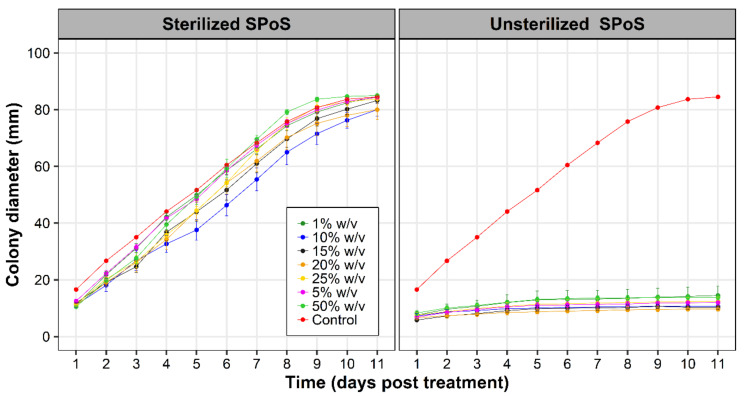
Mycelial growth of *Fusarium oxysporum* f. sp. *cubense* on potato dextrose agar (PDA) amended with varying concentrations (% *w*/*v*) of sterilized (**left** panel) and un-sterilized (**right** panel) filtrates of spent *Pleurotus ostreatus* substrate (SPoS).

No differences (*p* > 0.05) in *Foc* colony growth were observed between PDA amended with sterilized SPoS extracts of varying concentrations of 1%, 5%, 10%, 15%, 20%, 25%, and 50% (*w*/*v* of SPoS to sterile water) and the sterile water control (Figure 2 left panel). In contrast, *Foc* colony growth on PDA supplemented with unsterilized SPoS filtrates (concentrations as above) was significantly (*p* < 0.05) suppressed (Figure 2 right panel). No significant (*p* > 0.05) differences in *Foc* suppression were however observed between the different concentrations of the unsterilized SPoS filtrate.

### 3.3. Greenhouse Evaluation of Spent P. ostreatus Substrate (SpoS) against Fusarium oxysporum *f. sp.* cubense

**Figure 3:**
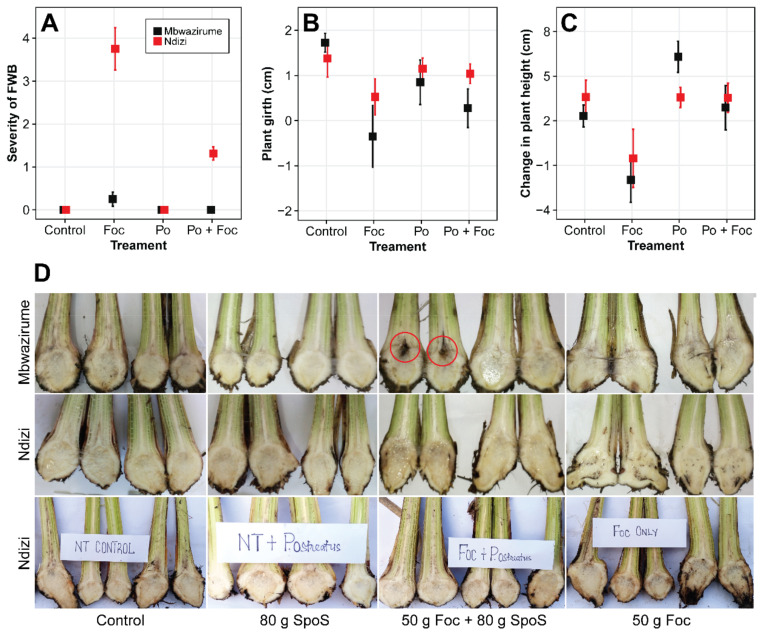
Severity of Fusarium wilt of banana (FWB) in corms (scale: 0 to 5) (**A**), pseudostem girth (**B**), plant height (**C**), and corm tissues appearance (**D**) of potted banana plants (resistant ‘Mbwazirume’ (AAA) and susceptible ‘Ndizi’ (AAB) cultivars) at 2.5 months after being subjected to different treatment combinations. The soils were sterilized and subjected to different treatments that included: a control in which the sterile soil was not amended, soils amended with 80 g of spent *P. ostreatus* substrate (SPoS), soils amended with 80 g SPoS and 50 g *Foc* colonized millet grain, and soils amended only with 50 g of *Foc* colonized grain. The zones circled in red show damage due to banana weevils.

The inoculation process using the *Foc* R1 isolate was efficient to cause FWB in both banana cultivars and allowed a clear cultivar discrimination regarding resistance levels (Figure 3A,D). In both the susceptible cultivar ‘Ndizi’ and resistant cultivar ‘Mbwazirume’, typical external symptoms of FWB were not observed at termination of the experiment, i.e., 2.5 months post inoculation.

When the effect of SPoS was analyzed, it was observed that a reduction on FWB severity occurred in both cultivars. In the susceptible cultivar, a significantly (*p* < 0.05) lower corm discoloration was observed in *Foc* inoculated soils amended with SPoS (score = 1.31) compared with the un-amended *Foc* inoculated soils (3.75) (Figure 3A,D). While severe corm discoloration was observed in ‘Ndizi’, only slight corm discoloration was observed in the resistant cultivar ‘Mbwazirume’. In the resistant cultivar, no corm discoloration was observed in the SPoS amended soil whereas a low (0.25) corm discoloration occurred in the *Foc* only soils (Figure 3A,D). However, corm discoloration in the *Foc* only soils for the resistant cultivar was not significantly different (*p* > 0.05) from that of the *Foc* inoculated soils amended with SPoS. For both the resistant and susceptible cultivar, no corm discoloration occurred in the control treatments (i.e., un-inoculated soils and soils amended with SPoS only).

The plants in *Foc* inoculated pots also had a retarded growth (Figure 3B,C), while *P. ostreatus* increased plant height and girth, even where both *Foc* and *P. ostreatus* had been jointly applied. No significant (*p* > 0.05) interactions were observed between the treatments and cultivars despite a higher increment in plant height and pseudostem girth in the control and *P. ostreatus* inoculated plants. Lower plant height and girth values were observed for ‘Mbwazirume’ plants treated with *Foc* despite resisting the pathogen (Figure 3B,C).

### 3.4. Suppression of Foc in ‘Ndizi’ Plants by SPoS in Naturally Foc-Infested Soils

Similar trends in corm damage to that in the artificially inoculated soils were observed in the ‘Ndizi’ plants planted in the naturally infected soils. *Foc* corm damage or discoloration was observed in both the un-amended naturally *Foc*-infested soils and the naturally *Foc*-infested soils amended with SPoS. However, SPoS significantly reduced the corm discoloration score (0.88) due to *Foc* when compared to 2.75 in the un-amended naturally *Foc-* infested treatment (Table 1). No corm damage occurred in plants treated with *P. ostreatus* only and the control treatments that had sterile soil.

In general, a decline in the aboveground growth of plants as shown by the changes in plant girth and height in *Foc* infected soils was observed, while only a retardation of growth occurred in the *P. ostreatus* treated plants in naturally infested soils (Table 1). Overall, no significant difference was observed between the treatments for plant girth, despite the decline in girth of plants in *Foc* infested soils. A significant difference (*p* = 0.004) in plant height was observed, with higher values visible for the sterile *P. ostreatus* amended soils and the control (Table 1). In contrast, dry root weight was higher in the *Foc* infested soil followed by the control and the least in the sterile soil with *P. ostreatus*, though no significant differences (*p* > 0.05) occurred between the treatments (Table 1).

## 4. Discussion

In this work, we show evidence, for the first time, that *P. ostreatus* can suppress *Foc* race 1 both in vitro and in greenhouse experiments. During the in vitro interactions *P. ostreatus* hyphae were observed to enlarge and become more vigorous at points of contact with *Foc* hyphae, suggesting that putative competition mechanisms are involved. The enlargement and increased vigor of *P. ostreatus* hyphae was subsequently followed by the degeneration of *Foc* on media, suggesting that, in addition to competition, antibiosis, and predation mechanisms could also be involved in *Foc* suppression by *P. ostreatus*. These interactions could also explain the lower number of *Foc* chlamydospores observed in the zone in which *Foc* degenerated due to interaction with *P. ostreatus*, relative to that in the zone adjacent to the point/zone of interaction. The higher number of *Foc* chlamydospores in this adjacent zone could be related to defense and survival mechanisms of *Foc* to overcome *P. ostreatus*. For example, in the interaction between phytobacterium *Ralstonia solanacearum* and the fungi *Fusarium fujikuroi* (causal agent of foolish seedling disease of rice), the bacterium has been reported to produce a lipopeptide (ralsolamycin) that triggers production of chlamydospores in the fungi [30].

Antifungal activity, from unsterilized substrates of *P. ostreatus* and other mushroom species, has been reported against other *formae specialis* of *F. oxysporum,* such as *F. o.* f. sp *lycopersici* in tomatoes [15], *F. o*. f. sp. *melonis* in melon crop [31], and *F. o* f. sp. *cepae, which causes* basal rot disease in shallot pots [32]. Our results also showed that the unsterilized filtrate of SPoS significantly inhibited the growth of *Foc,* even at low concentration (1% *w*/*v*).

The fact that the sterilized filtrate had no effect on the growth of *Foc* mycelia suggests that the suppressive effect of SPoS could be attributed to heat labile secondary metabolites, from *P. ostreatus* and inherent micro-organisms in the SPoS. *P. ostreatus* compounds, such as Pleurostrin, have been shown to have antifungal activity against *Fusarium oxysporum*, *Mycosphaerella arachidicola* and *Physalospora piricola* [33,34,35]. The substrate of *P. ostreatus* has also been reported to contain a wide diversity of microorganisms with strong antagonism toward pathogenic fungi [15,31]. Adedeji and Aduramigba [15] recovered *Trichoderma viridae*, fluorescent *Pseudomonas* spp., *Baccilus* spp., *Aspergillus terrus* and *Penicillum* spp., from spent substrate of *P. ostreatus* that had a strong antagonism to *Fol* [15]. Spent mushroom substrate teas have also been shown to suppress the phytopathogens *Phytophthora capsici* and *Phytophthora parasitica,* in vitro and in vivo [14]. Metabolomic studies and the purification of specific compounds from SPoS involved on *Foc* suppression, as well as mechanisms involved need further investigation.

In potted trials, suppression of *Foc*, shown by a lower corm damage in potted plants treated with *P. ostreatus*, was observed in both pre-sterilized artificially inoculated soils and naturally infested unsterilized soils. Failure of bio-control agents under natural field conditions due to the interactions with environmental and bio-physical factors has been a major constraint to their deployment. The success of *P. ostreatus* in naturally infested unsterilized soils suggests that *P. ostreatus* has the potential to succeed under field conditions. However, further studies to determine the effect of *P. ostreatus* on *Foc* under field conditions and different levels of management are still needed, for more concrete recommendations.

As expected, ‘Ndizi’ the *Foc* susceptible cultivar recorded a higher level of *Foc* damage. The resistant cultivar ‘Mbwazirume’ also registered some corm damage, albeit low. This cultivar and other EAH types (*Musa* AAA) have been reported to be immune or to resist *Foc* race 1 [24].

*P. ostreatus* spent substrate also increased overall aboveground biomass of the plants. Marin et al. [14] also reported spent mushroom substrate teas to promote plant growth. Increased plant vigor could have also improved the plants’ ability to resist *Foc* infection. Lower plant height and girth scores for the resistant ‘Mbwazirume’ cultivar can be attributed to banana weevil (*Cosmopolites sordidus*) damage that was observed in some of the assessed corm tissues. ‘Mbwazirume’ like other EAH banana cultivars is highly susceptible to banana weevils [36].

The root weight of plants was increased by *Foc* infection. This could be due to the formation of more roots to replace those lost or damaged by the infection. In beans, development of lateral roots above the initial sites of infection caused by *F. solani* f. sp. *phaseoli* infection and other root rot pathogens, such as *Pythium* spp. and *Rhizoctonia solani,* has been reported [37].

## 5. Conclusions

This study shows a high potential of the *P. ostreatus* spent substrate as part of the management practices to reduce the impact of FWB. Given the current increase in mushroom production in East Africa, its application could ensure an effective recycling of crop wastes and higher crop productivity, while reducing the burden of FWB. The fact that *P. ostreatus* will contribute to the incomes and nutrition of the farming communities makes its use a relatively cheap, environmentally sound, and farmer friendly option for sustainable management of FWB. Additional field studies under different disease pressure and environmental and management settings to evaluate the efficacy of *P. ostreatus* are recommended and ongoing. In addition, studies to elucidate the inhibition mechanisms of *P. ostreatus* (compounds), *P. ostreatus* spent substrate, and associated micro-organisms are ongoing. Studies on the efficacy of different *P. ostreatus* substrate types are also recommended.

## Figures and Tables

**Table 1:** Corm damage score (scale from 0 to 5; 0 = no damage, 5 = very severe damage) and change in pseudostem girth, height, and dry root weight of ‘Ndizi’ plants in (i) soils naturally infested with *Fusarium oxysporum* f. sp. *cubense* Race 1 (*Foc*), (ii) *Foc* infested soils amended with 80 g of spent *P. ostreatus* substrate (SPoS), (iii) sterile soils amended with SPoS, and (iv) an unamended sterile soil as control.

Treatments	Corm Damage Score (±sd)	Plant Girth (cm ± sd)	Plant Height (cm ± sd)	Dry Root Weight (g ± sd)
*Foc*-infested soil	2.75 ± 0.50	-0.40 ± 0.34	−0.90 ± 0.52	5.20 ± 1.41
*Foc*-infested soil + SPoS	0.88 ± 0.83	0.43 ± 0.69	0.74 ± 0.48	3.79 ± 1.30
Sterile soil + SPoS	0.00 ± 0.00	1.08 ± 1.42	2.38 ± 1.55	3.02 ± 1.81
Control (sterile soil)	0.00 ± 0.00	1.60 ± 1.80	2.28 ± 2.14	4.53 ± 1.55
*p* value	1.53e-05	0.1718	0.0040	0.2714

## Data Availability

The raw data supporting the conclusions of this manuscript will be made available by the authors, without undue reservation, to any qualified researcher.

## Supplementary Materials

The following are available online at https://www.mdpi.com/article/10.3390/jof7110946/s1, Figure S1: *Pleurotus ostreatus* garden showing healthy fruiting bodies, Figure S2: Overview of experimental setup to determine the in vitro interspecific interaction, antagonistic effect and mycoparasitism of *Pleurotus ostreatus* (*Po*) on *Fusarium oxysporum* f. sp. *cubense* (*Foc*).
