# Supplementary figures and images for: Spent Pleurotus ostreatus Substrate Has Potential for Managing Fusarium Wilt of Banana
Source: J Fungi (Basel). 2021 Nov 9;7(11):946. doi: 10.3390/jof7110946 (PMC8620842; doi:10.3390/jof7110946)

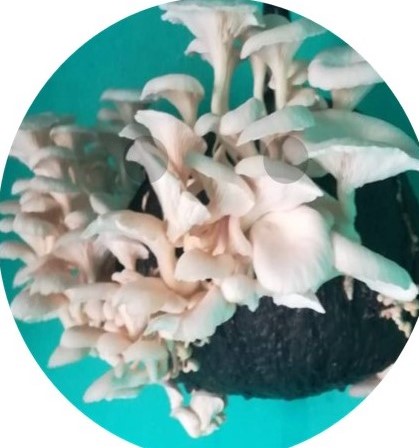

Supplement: Supplementary file 1 [file jof-07-00946-s001.zip › Supp figures/Supp_Figure 1_Po garden.jpg]

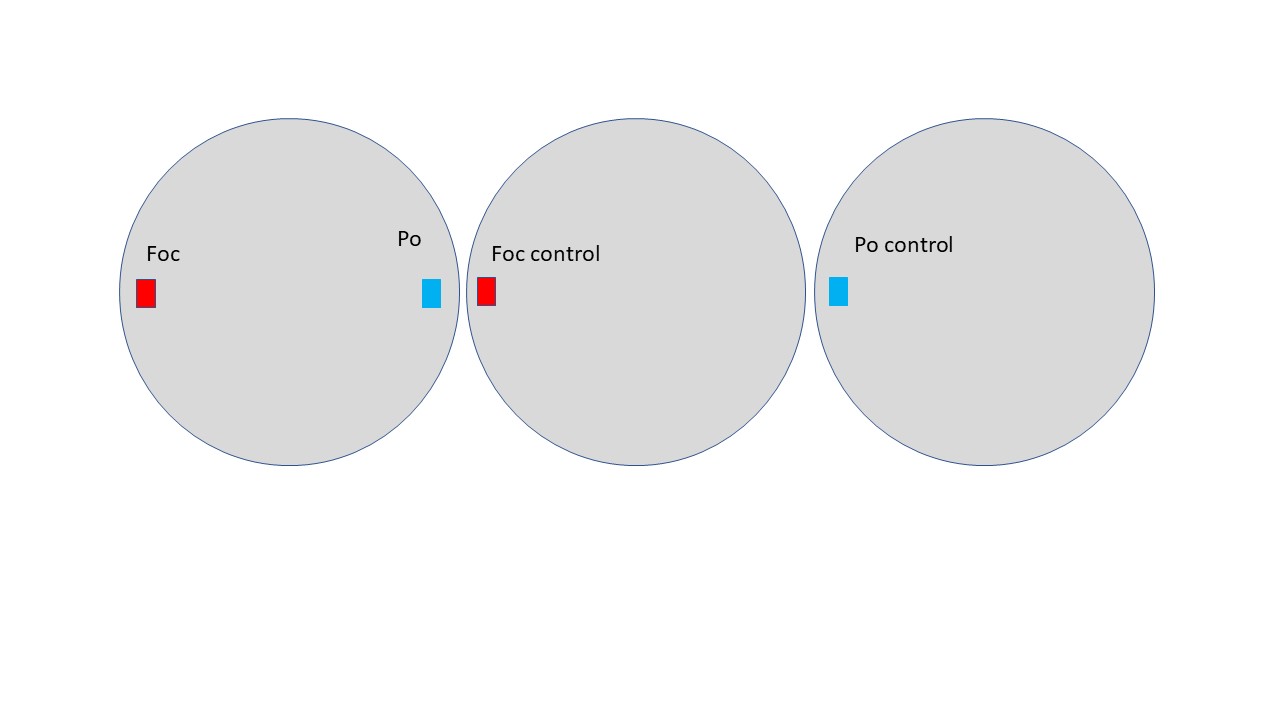

Supplement: Supplementary file 1 [file jof-07-00946-s001.zip › Supp figures/Supp_Figure 2 Co-culture plates.jpg]
